# Forced Degradation Testing as Complementary Tool for Biosimilarity Assessment

**DOI:** 10.3390/bioengineering6030062

**Published:** 2019-07-21

**Authors:** Yan Felix Karl Dyck, Daniel Rehm, Jan Felix Joseph, Karsten Winkler, Volker Sandig, Wolfgang Jabs, Maria Kristina Parr

**Affiliations:** 1 Department of Pharmaceutical & Medicinal Chemistry, Institute of Pharmacy, Freie Universität Berlin, Königin-Luise-Straße 2+4, 14195 Berlin, Germany; 2 Department of Life Sciences & Technology, Beuth Hochschule für Technik Berlin, Seestraße 64, 13347 Berlin, Germany; 3 ProBioGen AG, Goethestraße 54, 13086 Berlin, Germany; 4 Core Facility BioSupraMol, Institute of Pharmacy, Freie Universität Berlin, Königin-Luise-Str. 2+4, 14195 Berlin, Germany

**Keywords:** middle-up approach, liquid chromatography-mass spectrometry (LC-MS), QTOF-MS, biopharmaceuticals, forced stability testing, structure reactivity relationship, bevacizumab, infliximab, biosimilar

## Abstract

Oxidation of monoclonal antibodies (mAbs) can impact their efficacy and may therefore represent critical quality attributes (CQA) that require evaluation. To complement classical CQA, bevacizumab and infliximab were subjected to oxidative stress by H_2_O_2_ for 24, 48, or 72 h to probe their oxidation susceptibility. For investigation, a middle-up approach was used utilizing liquid chromatography hyphenated with mass spectrometry (LC-QTOF-MS). In both mAbs, the Fc/2 subunit was completely oxidized. Additional oxidations were found in the light chain (LC) and in the Fd’ subunit of infliximab, but not in bevacizumab. By direct comparison of methionine positions, the oxidized residues in infliximab were assigned to M55 in LC and M18 in Fd’. The forced oxidation approach was further exploited for comparison of respective biosimilar products. Both for bevacizumab and infliximab, comparison of posttranslational modification profiles demonstrated high similarity of the unstressed reference product (RP) and the biosimilar (BS). However, for bevacizumab, comparison after forced oxidation revealed a higher susceptibility of the BS compared to the RP. It may thus be considered a useful tool for biopharmaceutical engineering, biosimilarity assessment, as well as for quality control of protein drugs.

## 1. Introduction

As is generally the case for biopharmaceuticals, therapies with monoclonal antibodies (mAbs) are highly expensive, and therefore access is limited worldwide. When biopharmaceuticals run out of patent protection, biosimilars are developed as follow-on products [1]. For their approval, abbreviated procedures may be accepted by the authorities in analogy to the small molecule generics [2]. Physicochemical similarity plays a major role in the approval process. Critical quality attributes (CQA) have to be evaluated, which may impact pharmacological response [3].

Biosimilars offer a comparatively cheaper alternative to alleviate the burden that biopharmaceutical therapies pose to health care systems [4]. Currently, 11 biosimilar mAbs have been approved for five different blockbuster mAbs in the US and the EU [5]. Among these are biosimilars for Avastin (bevacizumab) and Remicade (infliximab).

Bevacizumab binds to vascular endothelial growth factor A (VEGF-A), preventing the activation of VEGF receptors on endothelial cells, which is necessary for their proliferation and migration. Thereby, bevacizumab inhibits angiogenesis, a process that is critical in the development of different tumors. Infliximab acts by binding to tumor necrosis factor-α (TNF-α), a cytokine that mediates inflammatory responses and modulation of the immune system. Infliximab is used for therapy of autoimmune and inflammatory diseases.

A therapeutic mAb product is a complex heterogenic mixture. While the same amino acid sequence is shared among all the mAb molecules in the mixture, different posttranslational modifications (PTM) are introduced during the production process. Its development and production therefore demand for special analytical methods [6,7,8].

One important PTM is oxidation, because it can have impact on the safety and efficacy of the mAb product. A close examination is mandatory in engineering and quality control of biopharmaceuticals. Thus, oxidations may be considered critical quality attributes (CQA), especially if they occur in the complementary determining regions (CDR), where they may impact the binding to the target antigen [9]. Oxidation also plays an important role if it occurs in the Fc part. Oxidations in this part can impact structure and stability [10] as well as the binding to protein A and protein G, relevant for downstream processing. Furthermore, a modified binding to neonatal Fc receptor for IgG (FcRn) may impact serum half-life [11,12,13]. Among several amino acids that can undergo oxidation, reactions of methionines have been extensively studied, because of their high susceptibility [14].

To investigate the susceptibility of a mAb towards oxidation, forced oxidation studies are performed in which exaggerated conditions are used to forcefully elicit oxidation. These studies are especially important in the identification of CQAs [15,16]. According to the International Council for Harmonisation of Technical Requirements for Pharmaceuticals for Human Use (ICH), stability testing of new drug substances and products should include long-term, intermediate, and accelerated stability testing (ICH Q1A) [17]. It is recommended to investigate the effect of temperature, humidity, oxidation, and photolysis on the drug substance. For accelerated oxidation, H_2_O_2_ or tert-butylhydroperoxid (tBHP) are reported to specifically target methionines [14,18]. In the same studies, 2,2-azobis(2-amidinopropane) dihydrochloride (AAPH) mainly targeted tryptophanes when additional free methionine was added to the reaction mixture. However, a recent study on one mAb showed that while H_2_O_2_ and tBHP exclusively led to methionine oxidation in the Fc, AAPH additionally oxidized both tryptophanes and methionines, which led to considerably different functional impacts, compared to the peroxides [19]. Thus, the specificity of AAPH for tryptophanes seems less clear.

Sources of oxidation, to which a mAb product is exposed, include peroxides, polysorbates, sugars, metals, air oxygen, and light [20]. Oxidation may occur during fermentation, purification, filling, and finishing, as well as during storage. It is thus considered as a critical degradation reaction. Therefore, the products oxidation profile depends on and can vary according to different production processes.

Reversed phase liquid chromatography (RPLC) is the preferred method for investigation of mAb oxidation on the physiochemical level because of its resolution, robustness, and compatibility with mass spectrometry [6]. The changed hydrophobic nature of oxidized species allows for their separation by RPLC and mass-spectrometric detection provides high selectivity.

The mAbs are analysed by LC-MS with different levels of structural resolution, and each level offers different advantages [21]. Determination of the mass of the intact mAb (also referred to as top level) is well suited for high-throughput applications, because it requires the least sample preparation and yields general information, such as verification of the expected mass and major modifications, such as the main glycoforms. As a complementary approach for detailed structural resolution, the mAb is digested into small peptides (bottom-up level). Also called peptide mapping, this approach reveals minor and less abundant modifications and enables their localization to specific peptides. However, because of elaborate and time-consuming sample preparation to avoid the artificial introduction of PTMs, this application only allows for low throughput [22]. In a third approach (middle up level), mAbs are enzymatically digested to yield the subunits fragment crystallizable region (Fc) and antigen-binding fragment (Fab). The subsequent reduction of disulfide bridges liberates the light chain (LC), the variable fragment of IgG heavy chain (Fd’), and the constant fragment of IgG heavy chain (Fc/2). Modifications can be localized to these three subunits. Thus, comparatively simple sample preparation and analysis make the middle-up approach a valuable complementary tool offering a compromise between high-throughput and detailed information.

The application of forced degradation with subsequent analysis on the subunit level has recently gained popularity. Among other degradation pathways such as deamidation, this approach has been used to study oxidation in mAbs, mostly elicited by chemical oxidation as well as by exposure to light [16,23,24,25,26,27]. Two biosimilarity studies of infliximab have included forced degradation in the biosimilarity assessment. Pisupati et al. used temperature and humidity stress [28]. However, no strong effect of oxidation was observed by peptide mapping. Kim et al. applied H_2_O_2_ on infliximab. By peptide mapping they showed methionine oxidations that were comparable between the reference product and the biosimilar [29]. Some studies have applied forced oxidation as well as other forced degradation treatments on bevacizumab and infliximab for analysis of aggregate formation, for binding assays and to support method validation [30,31,32,33]. However, as these studies were conducted on the intact mAbs, no structural information about PTMs introduced by forced degradation was provided.

In this study, biosimilars of bevacizumab and infliximab were subjected to forced oxidation using H_2_O_2_, together with their reference products, to support the biosimilar assessment. Analysis on the middle-up level was selected in order to provide detailed information about the susceptibility to oxidation of methionine residues. The results of the faster middle-up method are checked for consistency to published data obtained from the bottom-level [29].

## 2. Materials and Methods

### 2.1. Chemicals and Consumables

Hydrogen peroxide (H_2_O_2_) solution (30% (w/w) in H_2_O), tris(2-carboxyethyl)phosphine (TCEP, 0.5 M, pH 7.0 aqueous solution), and 2-(*N*-morpholino)ethanesulfonic acid monohydrate (MES monohydrate) were obtained from Sigma-Aldrich (Steinheim, Germany). Sodium azide (NaN_3_) and potassium dihydrogen phosphate (KH_2_PO_4_) were obtained from Merck Millipore (Darmstadt, Germany). Acetonitrile (ACN) and formic acid were obtained from Fisher Scientific (Schwerte, Germany). Di-sodium hydrogen phosphate dihydrate (Na_2_HPO_4_·2H_2_O) was purchased from Bernd Kraft GmbH (Duisburg, Germany), and guanidine-HCl was from AppliChem (Darmstadt, Germany). All purchased chemicals were of highest purity. Ultrapure water was obtained using LaboStar 2-DI/UV system (SG Wasseraufbereitung und Regeneration GmbH, Barsbüttel, Germany) equipped with LC-Pak Polisher and a 0.22-μm membrane point-of-use cartridge (Millipak). Mass calibration reference masses (HP-921, HP-1821, HP-2421) were from Agilent (Waldbronn, Germany). IdeS enzyme and Vivaspin 500 ultrafiltration spin columns were purchased from Genovis (Lund, Sweden) and Sartorius (Göttingen, Germany), respectively.

Phosphate buffer of pH 6.5 (Sørensen’s phosphate buffer) was prepared freshly by mixing 6.87 volumes of a 66.71 mM KH_2_PO_4_ stock solution with 3.13 volumes of a 66.72 mM Na_2_HPO_4_·2H_2_O stock solution. MES buffer solution of pH 6.5 was 10 mM.

The bevacizumab reference product (RP) Avastin (lot B8502H09) from Roche (Basel, Switzerland) and the infliximab RP Remicade (lot 9RMKA60302) from Janssen Biologics B.V. (Leiden, The Netherlands) were used for this study. The biosimilar (BS) antibodies were produced at ProBioGen AG. The biosimilars were expressed in chinese hamster ovary cell culture, purified from the supernatant by protein A and eluted at a low pH. Bevacizumab biosimilar was further kept at a low pH for virus inactivation. Both BS antibodies were buffer-exchanged with the same buffer as the RP. Other purification steps that would be performed for clinical material were not applied.

### 2.2. Generation of Stressed mAb Samples

Forced oxidation was performed by incubation of RP and BS at a final concentration of 0.6 µg/µL antibody, 0.05% (w/v) H_2_O_2_, 0.1% (w/v) NaN_3_ and phosphate buffer (pH 6.5 at a final dilution of 1:4) at 37 °C for 24 h, 48 h, or 72 h after thorough vortexing (Table 1). Control samples, prepared using water instead of H_2_O_2,_ were treated similarly. All experimental stress conditions were applied to the biopharmaceuticals as independent triplicates. After forced oxidation, mAbs were five times buffer exchanged with 400 µL MES buffer by centrifugation in an ultrafiltration column (molecular weight cut off = 10 kDa) and then diluted to 1 µg/µL in MES buffer. Protein concentration was determined by measuring the absorbance at 280 nm on a Nanodrop ND-1000 spectrophotometer (NanoDrop Technologies, Wilmington, DE, USA).

### 2.3. Digestion and Reduction of mAb Samples

Stressed mAb samples were digested at a final concentration of 1 µg/µL in MES buffer with IdeS at 1 unit enzyme/1 µg mAb. To yield the Fc/2, Fd’, and LC subunits, 100 µg of mAb were buffer-exchanged and adjusted to a final volume of 50 µL MES buffer (β_mAb_ = 2 µg/µL). IdeS (10 µL of a stock solution, β_stock_ = 10 units/µL) was added and the final volume was adjusted to 100 µL by addition of MES buffer (β_mAb_ = 1 mg/µL; β_IdeS_ = 1 unit/µL). Digestion was achieved within 30 min at 37 °C. For reduction, 500 µL of guanidine-HCl (8 M solution) and 66 µL of TCEP solution (500 mM) were added to result in a final volume of 666 µL. Thus, the composition of the final mixture expressed in volume percent is: mAb digest (15%), 8 M guanidine-HCl (75%), and 500 mM TCEP (10%). After vortexing samples were incubated at ambient temperature for 1 h to complete reduction of the disulfide bonds.

### 2.4. Liquid Chromatography and Mass Spectrometry

Samples were analysed using a 1290 Infinity II UHPLC system coupled to a 6550 iFunnel Q-TOF mass spectrometer with an electrospray ionization source (Agilent, Waldbronn, Germany). Aliquots of the sample (0.75 µg) were injected onto a reversed phase C4 column (Aquity UPLC Protein BEH C4 Column, 300 Å, 1.7 µm, 2.1 mm, 100 mm, Waters, Manchester, UK) and the column temperature was maintained at 80 °C. Mobile phase A was 0.1% (*v*/*v*) formic acid in water, and mobile phase B was 0.1% (*v*/*v*) formic acid in ACN. The flow rate was 0.5 mL/min. The complete run consisted of 1 min isocratic elution (20% solvent B), followed by a 40 min gradient (20% to 32% solvent B), a subsequent purge step (quick rise to isocratic elution at 95% solvent B), and a final 5 min reequilibration step (quick decrease to isocratic elution at 20% solvent B).

Mass spectrometer settings included a mass range of *m*/*z* 600 to *m*/*z* 3,200; nebulizer: 2.8 bar, drying gas flow: 14 L/min; drying gas temperature: 290 °C; sheath gas flow: 12 L/min; sheath gas temperature: 375 °C; fragmentor voltage: 400 V. Spectra were acquired at 2 spectra/s. Internal mass calibration was achieved by continuously spraying of internal reference compounds through the reference nebulizer.

Data acquisition was controlled with the MassHunter software (Agilent, Santa Clara, CA, USA).

### 2.5. Data Processing

For the identification of species, an averaged raw spectrum was generated for each chromatographic peak and deconvoluted using the maximum entropy algorithm in Bioconfirm software (Agilent). Deconvoluted masses were matched to expected masses calculated based on the amino acid sequence of the antibody and variable posttranslational modifications (with ±70 ppm match tolerance).

Semiquantification was based on extracted ion chromatograms (EIC). For each species, the most intensive *m*/*z* charge state was used with an *m*/*z* tolerance of ±0.01, and the intensity (I) of the resulting EIC peak was used. The relative amount of the 3-fold oxidized (3ox) species was calculated according to:

$$a_{r}=\frac{I_{3ox}}{I_{0ox}+I_{3ox}}.$$

## 3. Results

### 3.1. Subunit Mass and PTMs of Unstressed Bevacizumab and Infliximab

For both biopharmaceuticals under investigation, i.e., bevacizumab and infliximab, IdeS digestion and reduction resulted in the separation of the antibody subunits as expected. The units were separated by liquid chromatography and detected in the MS as individual signals.

In biosimilar comparison, the amino acid sequence is the most basic criterion. The measured subunit masses matched with those calculated from the amino acid sequence, for the RP as well as for the BS for both mAbs. In case of both mAbs, no differences were observed between the respective RP and the BS, except that in infliximab RP, c-terminal lysine clipping was incomplete, whereas in the BS it was almost complete.

**Figure 1:**
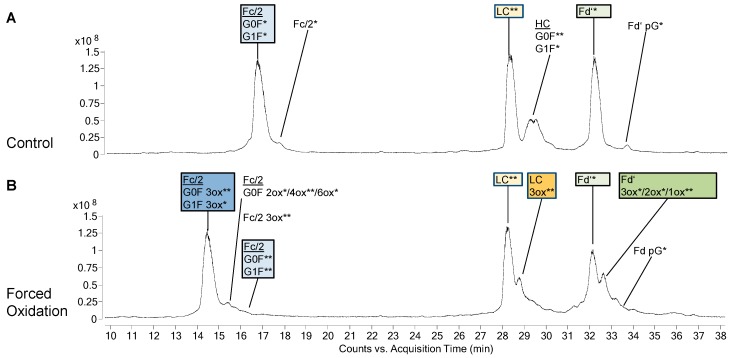
Species identified in biosimilar (BS) of bevacizumab after 48 h of control treatment (**A**) or forced oxidation (**B**). Annotations show the different protein species and their modifications in the chromatographic peaks as identified by their mass. The mass difference between the observed mass and the theoretical value is indicated as follows: 0 to 0.5 Da = *, 0.6 to 1.5 Da = **, 1.6 to 4.0 Da = ***. Ox, oxidations; pG, pyroglutamate modification. Coeluting species within one annotation are listed in order of spectrum intensity.

**Figure 2:**
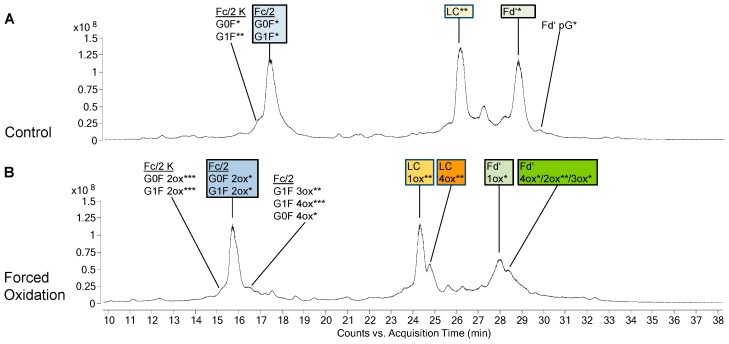
Species identified in biosimilar (BS) of infliximab after 48 h of control treatment (**A**) or forced oxidation (**B**). Annotations show the different protein species and their modifications in the chromatographic peaks as identified by their mass. The mass difference between the observed mass and the theoretical value is indicated as follows: 0 to 0.5 Da = *, 0.6 to 1.5 Da = **, 1.6 to 4.0 Da = ***. K, additional c-terminal lysine; Ox, oxidations; pG, pyroglutamate modification. Coeluting species within one annotation are listed in order of spectrum intensity.

PTM profiles of the unstressed mAbs were analysed, as PTMs represent an important criterion in biosimilarity assessment. Figure 1A and Figure 2A show the total ion chromatograms (TIC) of the 48 h control of bevacizumab BS and infliximab BS, respectively. As expected, both mAbs did not show any PTMs in the LC. The PTM profile of unstressed bevacizumab BS included Fc glycans G0F and G1F as well as small amounts of unglycosylated Fc/2 and Fd’ pyroglutamate (Figure 1A). Also for infliximab BS, Fc glycans G0F and G1F and a small amount of Fd’ pyroglutamate were found (Figure 2A). Low levels of oxidized Fc/2 species were found in both mAbs. Especially infliximab displayed low levels of oxidized LC and Fd’ species, whereas in bevacizumab, these were almost absent. In both mAbs, a noticeable chromatographic peak eluted between the LC and the Fd’. In case of bevacizumab, this peak was identified as the undigested heavy chain. In infliximab this peak contained not only the heavy chain but also several masses that presumably represented fragments. No difference was observed between the different time points of control treatment (without H_2_O_2_), suggesting that the control treatment did not exert any effect on the sample. In both mAbs, analysis of the unstressed samples showed a high degree of similarity between BS and RP.

### 3.2. Forced Oxidation

For forced oxidation, samples were treated with H_2_O_2_ for 24 h, 48 h, and 72 h. Analogously, to the above-mentioned analysis of the control samples, the chromatographic profiles of the BS after forced oxidation closely resembled that of the RP.

In bevacizumab, the most abundant species of Fc/2 after forced oxidation was the 3-fold oxidized (3ox) species, while its LC and Fd’ mostly remained unoxidized (Figure 1B). When infliximab was oxidized, the most abundant Fc/2 species was the 2ox species (Figure 2B). Furthermore, its LC had been completely converted with 1ox LC representing the most abundant species. Similar to the findings for the LC, the biggest part of infliximab Fd’ was found as 1ox. Concerning these main oxidation states, no differences were observed between BS and RP of bevacizumab and infliximab.

In addition to the main oxidized species described above, additional species with higher oxidation states appeared. In case of the LC and Fd’, these higher oxidized species eluted as peaks that were not fully separated from the main oxidized species. In bevacizumab, in addition to the main species of 3ox Fc/2, unoxidized LC and unoxidized Fd’ the next most abundant species were 4ox Fc/2, 3ox LC and 3ox Fd’, respectively (Figure 1B). In infliximab, in addition to the main species of 2ox Fc/2, 1ox LC, and 1ox Fd’, the next most abundant species were 3ox Fc/2, 4ox LC, and 4ox Fd’, respectively (Figure 2B).

Some minor differences were found between BS and RP with regard to the oxidation states. In bevacizumab, small amounts of higher oxidation states of Fc/2 (4ox, 6ox) were only found in the BS. Likewise, in infliximab, only the BS displayed small amounts of higher oxidation states of Fc/2 (3ox, 4ox) and smaller amounts of Fd’ intermediate oxidation states (2ox, 3ox) were only found in the BS, whereas these were virtually absent in the RP.

### 3.3. Time Course of Forced Oxidation for Comparison of BS and RP

**Figure 3:**
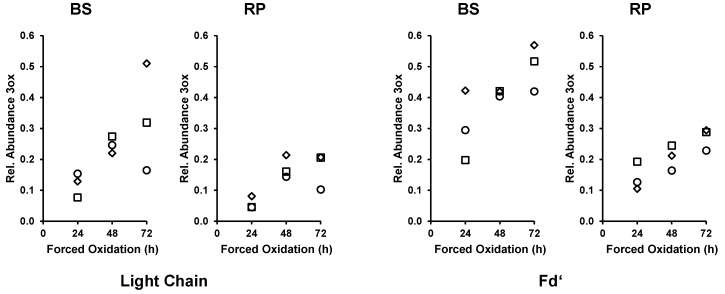
Time course analysis of bevacizumab biosimilar (BS) and reference product (RP) in forced oxidation samples. EICs were generated for light chain (LC) 0ox, LC 3ox, Fd’ 0ox and Fd’ 3ox and height of the EIC peak was measured. The rel. abundance 3ox was calculated using the equation given in Section 2.5. Square, circle, and diamond each represent one experimental repetition. Ox, oxidations.

While the Fc/2 of bevacizumab was completely converted from the unoxidized to the 3ox species, the bigger part of the LC and Fd’ remained unoxidized. Analysis of the TICs of bevacizumab (as shown in Figure 1B) of the 24 h, 48 h, and 72 h forced oxidation experiment indicated an ongoing shift of LC and Fd’ species from their main species (0ox) to the higher oxidation states. To elucidate and monitor the progression of the oxidation reaction, EICs were generated for the unoxidized species (LC 0ox and Fd’ 0ox) and for the corresponding oxidized species (LC 3ox and Fd’ 3ox as the most abundant oxidized species). The relative abundance of 3ox species increased with longer forced degradation treatment, both for the LC and the Fd’, in BS as well as in the RP (Figure 3). This analysis of the forced oxidation samples showed that the ongoing conversion of both the bevacizumab LC and Fd’ was stronger in the BS than in the RP.

## 4. Discussion

### 4.1. PTMs in Unstressed Samples

The PTM profiles of BS and RP were compared in unstressed samples of bevacizumab and infliximab (Figure 1A and Figure 2A). All samples were processed on the middle-up level and thus, glycosylated Fc/2, and unmodified LC and Fd were observed as main species. RP and BS showed highly similar PTM profiles in case of both mAbs, with the exception that in infliximab, lysine clipping was found to be different for RP and BS. The presence of c-terminal lysine does not alter the biological activity, but it should be monitored as an indicator for a consistent production process [6,34]. Additional species of low abundance included N-terminal pyroglutamate in both mAbs and unglycosylated Fc/2 in bevacizumab. These results underline the value of the middle-up approach for PTM investigation. A similar approach was also reported by Zhang et al. in another mAb study [16]. Interestingly, the unstressed infliximab samples displayed slightly higher initial levels of oxidized species than those of bevacizumab, as will be discussed later. Biosimilar comparison also showed high similarity with the RP in terms of the amount of oxidized species.

### 4.2. Subunit Analysis after Forced Oxidation

**Figure 4:**
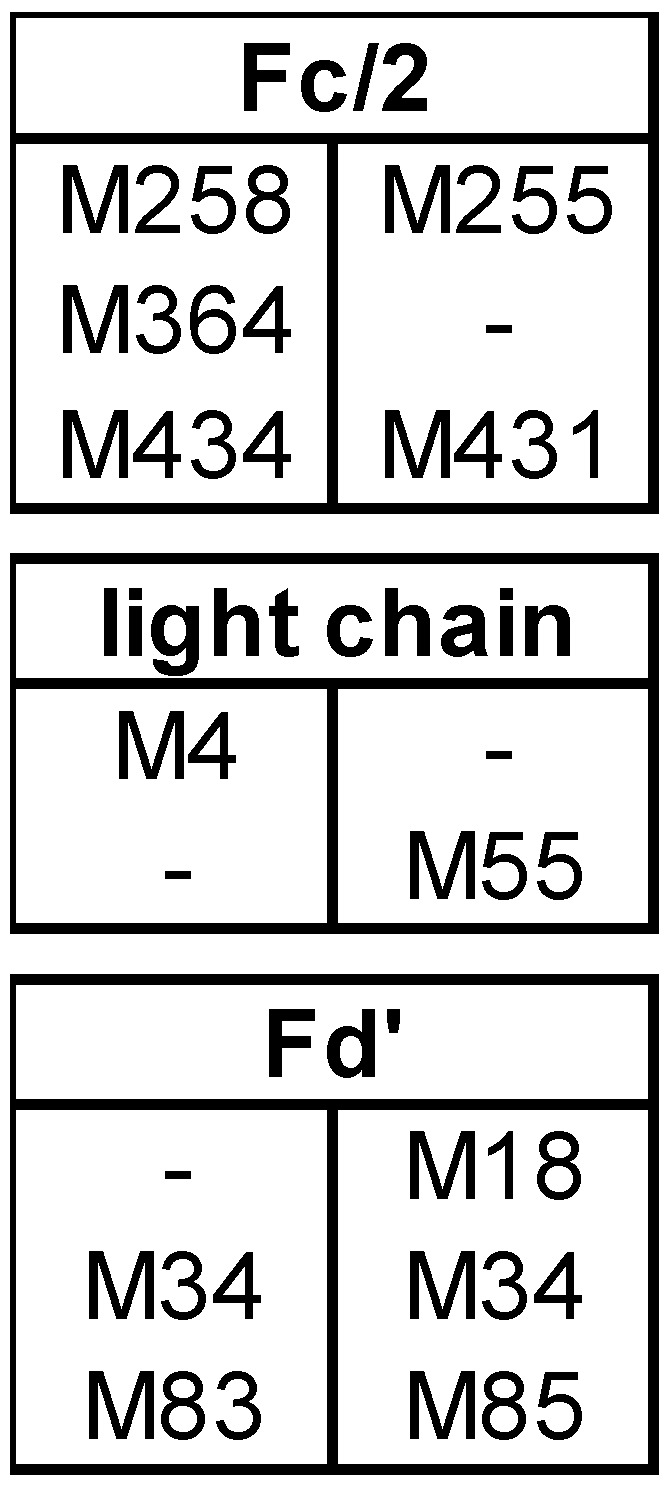
Methionine (M) positions for bevacizumab (left column) and infliximab (right column).

The results presented in our study can help in the identification of methionines that are prone to oxidation and which thus may represent potential CQA. The amount of oxidation that was predominantly observed for each subunit (here designated as most abundant species of the subunit) after forced oxidation was compared between bevacizumab and infliximab as both mAbs differ in the composition and position of methionines in the Fc and the Fab subunits (Figure 4).

The Fc/2 subunit harbors the two conserved methionines M252 and M428 and can additionally contain M358 (EU numbering [35], methionine positions in bevacizumab and infliximab are slightly different as shown in Figure 4). As M252 and M428 are surface exposed, they display a higher susceptibility towards oxidation [11,26,36]. Our data on bevacizumab oxidation show the most abundant species of Fc/2 as 3ox (Figure 1B), while in infliximab, the most abundant Fc/2 species was the 2ox species (Figure 2B). The methionine residues M252 and M428 are present in both mAbs, whereas bevacizumab additionally contains M358. These results suggest that all methionines of Fc/2 subunit become oxidized. Studies on other mAbs containing the same three Fc/2 methionine positions demonstrated that all three residues become highly oxidized when subjected to H_2_O_2_ [18,19]. When tBHP was used, M358 was found less oxidized than the two-surface exposed methionines [11,19,23]. Shah et al. compared different oxidative reagents and showed that for M358 H_2_O_2_ led to stronger oxidation than tBHP [19]. Kim et al. subjected infliximab to 0.1% H_2_O_2_ at 5 °C for up to 6 h [29]. Utilizing peptide mapping they found that M255 was highly oxidized, whereas M431 was not oxidized. In contrast, our data support the oxidation of M431 as well. Most likely the prolonged incubation time and higher temperature led to higher progression of oxidation in our study.

Concerning the Fab part, bevacizumab and infliximab behaved very differently. In bevacizumab, the LC and the Fd’ remained mostly unoxidized (Figure 1B). In contrast to the results of bevacizumab, in infliximab the LC and the Fd’ were completely converted to oxidized forms, with 1ox representing the most abundant form (Figure 2B).

Both mAbs contain one methionine in the LC, however at different positions, bevacizumab at M4 and infliximab at M55 (Figure 4). The finding that the bevacizumab LC was mostly unoxidized whereas the infliximab LC became mostly 1-fold oxidized, suggests that M4 is not reagent accessible, whereas M55 can be readily oxidized. Our finding of unoxidized M4 is in accordance with the results of Folzer et al., in which the only LC methionine, M4, remained mostly unoxidized [18]. Furthermore, our finding of oxidized LC M55 in infliximab is supported by the results of peptide mapping reported by Kim et al., who showed an intermediate level of M55 oxidation in infliximab, using milder conditions for forced oxidation [29].

The two methionine residues of the Fd’ subunit, M34 and M83 (M83 in bevacizumab corresponding to M85 in infliximab), are present in both mAbs. Additionally, infliximab contains M18, which is not present in bevacizumab (Figure 4). While the Fd’ subunit of bevacizumab remained mostly unoxidized, that of infliximab was completely transformed with 1ox Fd’ representing the most abundant oxidation state. This suggests that the 1ox Fd’ subunit in infliximab was generated by oxidation of M18. This is supported by literature reports that M34 and M83 undergo only slight oxidation upon forced degradation testing, despite the location of M34 in or adjacent to heavy chain CDR1 [13,18,23,37,38]. Our finding of oxidized M18 in Fd’ is also in accordance with the peptide mapping results of Kim et al, who showed that M18 was oxidized, whereas M34 and M85 were not oxidized in infliximab [29].

In summary, the comparison of bevacizumab and infliximab after forced oxidation suggested that in addition to the conserved and oxidation prone Fc/2 methionines of both mAbs, LC M55 and Fd’ M18 of infliximab are highly susceptible to oxidation.

Small levels of oxidized species were found in the unstressed control samples of infliximab, but were basically absent in bevacizumab. This is probably due to the oxidation prone methionines found in infliximab LC and Fd’, which may undergo slight oxidation during production or storage.

Additionally, lower amounts of higher oxidation states were found for each subunit of the stressed samples, i.e., 4ox Fc/2, 3ox LC and 3ox Fd’ in bevacizumab and 3ox Fc/2, 4ox LC and 4ox Fd’ in infliximab (Figure 1B and Figure 2B). These may occur on less solvent-exposed and thus slowly oxidizing methionines [13,18,23,37]. Another explanation may be the unspecific oxidation of other amino acids like tryptophanes that has also been observed elsewhere and that might be enhanced due to the harsh conditions used within this study [14,18].

### 4.3. CQA Considerations

The finding of oxidation susceptible methionines in Fc and Fab indicates that these residues may represent CQA. For a full CQA assessment, structure-function relationships need to be established. However, including evidence from literature can initially help to estimate the criticality of the observed oxidations. A risk assessment based on the evaluation of the impact of oxidation on functional properties of the mAbs is strongly recommended for the establishment of acceptance criteria.

Oxidation of the conserved methionines in Fc has been extensively studied, as they are present in all therapeutic mAbs of the IgG1 class, which represents the majority of the marketed mAbs [23]. The resulting effects include decreased binding to Protein A and Protein G, which is used for mAb purification and can be used in different assays [11,12]. Also, a reduced binding to FcRn as well as to Fc-gamma receptors (FcγR) was demonstrated. Binding to FcRn contributes to the extended mAb circulation half-life while binding to FcγR is responsible for effector functions [11,19,39]. In case of bevacizumab, FcγR mediated effector functions are reported not to play a biological role, whereas for infliximab, antibody-dependent cell-mediated cytotoxicity (ADCC) may contribute in the activity in inflammatory bowel disease. Both for bevacizumab and infliximab, FcγR binding and related functional tests are part of the biosimilarity assessment [34,40]. Together, the known impact of oxidation indicates the role of the conserved Fc methionines as possible CQA.

While bevacizumab did not show strong levels of oxidations in the Fab, infliximab showed strong oxidation in both, the LC and in the Fd’ subunit. Our findings suggest that in infliximab M18 of the Fd’ and M55 of the LC become oxidized. As M55 is located in the CDR2, the oxidation of this residue may directly impact the target binding [38]. Oxidation of methionines as well as of trypthophanes in the CDRs can result in destabilisation of Fab, increased aggregation as well as decreased target binding [9,19,41]. Pisupati et al. performed a comparability study between infliximab and the biosimilar Remsima [28]. Both the RP and the BS were analysed after subjection to elevated temperature and humidity stress. The stress resulted in decreased TNF neutralizing ability which could be related to amino acid modifications in the CDRs such as LC M55 and HC M34. Thus, oxidation of infliximab at M55 may represent a CQA. M18 is located outside of the CDRs [38]. Still, the impact of M18 oxidation on properties such as aggregation tendency or target binding should be examined [19]. Thus, the findings of oxidation prone methionines in the Fc of both mAbs and in the Fab of infliximab are important for further investigations, such as on binding to FcRn and FcγR, as well as target binding. Such investigations are mandatory to assess the functional implications of the detected modifications.

### 4.4. Different Susceptibility of Bevacizumab BS and RP during Forced Oxidation

Regarding the biosimilar comparison, we intended to use harsh oxidation conditions, because milder conditions might not be sufficient to amplify initial differences between the BS and the RP. Our results demonstrate that the use of H_2_O_2_, which is a strong oxidant specific for methionines, is suitable for this purpose. It has been shown that different oxidative agents lead to different effects. In a study comparing H_2_O_2_, tBHP and AAPH for oxidation of one mAb, the peroxides strongly and almost exclusively oxidized the three Fc methionines [19]. AAPH additionally led to a smaller extend of Fab CDR oxidation, namely to one methionine and one tryptophan. Even though these residues were oxidized only to approximately 5%, a huge effect of the AAPH oxidation on the mAbs function such as ADCC, target binding, and related target cell proliferation was observed. Therefore, the use of tBHP and AAPH is addressed to future studies to yield a broader picture of oxidative degradation. The time course comparison of the two bevacizumab mAbs under forced oxidation showed an increased relative abundance of higher oxidation states in LC (3ox) and Fd’ (3ox) of the BS compared to the RP. Moreover, small amounts of higher oxidation states of Fc/2 (4ox, 6ox) were only observed in the BS. However, one should keep in mind that forced oxidation conditions over-exaggerate differences in the products. Therefore, Pisupati et al. recommended to interpret the results with caution [28]. Nevertheless, the observed results may point to differences in the unstressed products, which could initiate further oxidation. Many factors in the production process are known to contribute to oxidation, including excipients and impurities such as sugars, polysorbates, peroxides, and metals, as well as light [19,20]. The production process of a BS is always different from that of the RP. Different raw materials can be one possible reason for the observed discrepancy in oxidation levels after forced degradation. For example, the formulation of bevacizumab and infliximab contains polysorbate 20 and polysorbate 80, respectively. Polysorbates are a source of hydroperoxides which in turn can lead to oxidations in the mAb [42]. The forced oxidation studies presented here can help to identify and prevent sources of oxidation for example during screening of different excipients.

Here we applied H_2_O_2_ for forced oxidation to mAbs and performed analysis on the subunit level. The comparison of the different methionine positions between bevacizumab and infliximab not only allowed to localize the oxidations to the subunits, but even indicated which of the methionine positions were affected. Thus, the subunit approach reveals more relevant structural information than the analysis of intact mAbs. Peptide mapping generally provides more specific information but bears a considerable risk for artificial introduction of modifications, and hence requires comprehensive and time-consuming method optimization. The good accordance of the results presented here for infliximab with published peptide mapping results of this mAb underline the usefulness of the subunit analysis as a fast alternative for these types of study.

## Figures and Tables

**Table 1:** Overview of experiments. The reference product (RP) and biosimilar (BS) version of two mAbs (bevacizumab and infliximab) were subjected to forced oxidation (oxi) and control (con) treatment. Each experiment was performed as independent triplicate.

mAb	Product	Duration of Forced Oxidation or Control Treatment			
		0 h	24 h	48 h	72 h
**Bevacizumab**	RP	con -	con oxi	con oxi	con oxi
	BS	con -	con oxi	con oxi	con oxi
**Infliximab**	RP	con -	con oxi	con oxi	con oxi
	BS	con -	con oxi	con oxi	con oxi
